# Editorial: Exploration of genetic variation, drug response, and interactions between gastrointestinal disorders and other diseases

**DOI:** 10.3389/fphar.2023.1287441

**Published:** 2023-09-13

**Authors:** Shuangshuang Tong, Yanlin Lyu, Ruibang Luo, Felix Leung, Weihong Sha, Hao Chen

**Affiliations:** ^1^ Department of Gastroenterology, Guangdong Provincial People’s Hospital, Guangdong Academy of Medical Sciences, Southern Medical University, Guangzhou, China; ^2^ The Second School of Clinical Medicine, Southern Medical University, Guangzhou, China; ^3^ Shantou University Medical College, Shantou University, Shantou, China; ^4^ Department of Computer Science, The University of Hong Kong, Hong Kong, Hong Kong SAR, China; ^5^ Sepulveda Ambulatory Care Center, VA Greater Los Angeles Healthcare System, Los Angeles, CA, United States; ^6^ University of California Los Angeles David Geffen School of Medicine, Los Angeles, CA, United States

**Keywords:** genetic variation, drug response, interaction, gastrointestinal diseases, pathogenesis

The global prevalence of gastrointestinal disorders is increasing, resulting in considerable suffering and significant healthcare costs (Peery et al., 2022). Multiple factors, such as genetic variation and pharmacological agents, have been confirmed to play a role in the pathogenesis of gastrointestinal diseases (Camilleri, 2012; Laiyemo and Abreu, 2015; Valle et al., 2019). In recent years, with the development of genomic sequencing technologies, the influence of genetic variation has become the focus of medical research. Exploring the role of genetic variation can shed light on the development, progression, and prognosis of numerous diseases, clarify the differences in individual responses to drugs and understand the interactions between diseases. In this Research Topic, diverse studies contributed to the exploration of genetic variation, drug response, and interactions between gastrointestinal disorders and other diseases, and yielded new insights into the genetic basis of complex diseases and pharmacological actions.

With the advance of microbiome genetic variation analysis, the research on the human microbiome and its relationship with various traits has garnered significant attention (Garud and Pollard, 2020; Adiliaghdam et al., 2022). Recently, Sun et al. demonstrated the connection between one specific strain of gut microbiota *Bacteroides dorei* (BDX-01) and the development of acute colitis with dextran sulfate sodium induced colitis mice model and various cell models. In this study, researchers explored the detailed mechanism of the beneficial effect of BDX-01 on colitis through a variety of methods such as genome sequencing, enzyme activity analysis, targeted metabolomics, and animal experiment. Moreover, compared with mesalazine, the BDX-01 was shown to be more effective in improving colitis, providing a promising probiotic agent for management of the ulcerative colitis. Nonetheless, previous studies have been controversial on the role of *Bacteroides dorei* in different diseases (Zhang, Osaka and Tsuneda, 2015; Yao et al., 2018; Yoshida et al., 2018; Song et al., 2021), which suggested a disease specific probiotic property of B. *dorei*. The capacity of a particular microbiota to elicit specific epigenomic alterations in hosts has unveiled the complexity in the relationship between genetic variation and phenotypes. Given the unpredictable responses of each disease with different genetic background, it is necessary to thoroughly evaluate the safety before using a new microbial preparation.

While focusing the safety of the agents under development, the side effect of the existing drugs cannot be ignored. Take interleukin (IL)-17 inhibitors as an example; they have showed favorable therapeutic effects on most autoimmune diseases, but may have adverse effects on the gastrointestinal tract. Deng et al. comprehensively assessed the side effect of IL-17 inhibitor therapy on the onset of inflammatory bowel disease (IBD) based on literature review and database analysis. By integrating the real-world data including symptoms, laboratory indexes and histopathological examinations, researchers provided supportive evidence of the adverse gastrointestinal events caused by IL-17 inhibitor therapy. All three anti-IL-17 drugs analyzed exhibited different effects on the onset or exacerbation of IBD, which emphasized the importance of understanding the discrepancy in pharmacology of each drug and monitoring the side effect of the drugs in clinical practice. Besides, it was reported that IBD might overlap with several autoimmune diseases, such as psoriasis and ankylosing spondylitis, with several shared loci identified in a genome-wide association study (Jostins et al., 2012; Fieldhouse et al., 2020). However, these mentioned diseases exhibited completely different responses to the IL-17 inhibitors (Burisch et al., 2020; Gossec et al., 2020; Ramiro et al., 2023), which highlights the necessity to further analyze the interactions between IBD and rheumatic immune diseases.

As one of the most widely prescribed drugs, proton-pump inhibitors (PPIs) have been associated with growing concerning adverse reactions. Seah et al. conducted a retrospective cohort study to investigate the potential PPI-related hypomagnesemia in a single tertiary center. The research indicated that long-term use of PPIs could causally promote the development of severe hypomagnesemia, and higher doses of PPI would amplify the causal link. These findings remind clinicians to contemplate administering the minimal necessary dosage for patients and be cautious about the rare but severe adverse effect of PPIs. Genetic susceptibility to hypomagnesemia might contribute to individual variability in responses to PPIs. Hess et al. (2017) has identified two single nucleotide polymorphisms, rs3750425 and rs2274924, in individuals with PPI-associated hypomagnesemia. Thus, it is promising to establish a predictive model for adverse drug events by incorporating genetic and pertinent clinical data in the future.

Understanding the biological pathways of diseases is crucial for predicting the disease response, identifying pharmacological targets, and developing more appropriate therapy. Recently, Jiang et al. established a HIPPO signaling-related signature based on the Cancer Genome Atlas cohort and validated the prognostic efficacy in the Gene Expression Omnibus cohort. Previous research primarily concentrated on the impact of the Hippo pathway on gastric cancer progression, without examining its effect on the prognosis of gastric cancer. The risk signature proposed in this study was novel and superior to other existed clinical risk factors in predicting overall survival of gastric cancer. By adding the drug responses of patients to immunotherapy and chemotherapy, the constructed prognostic nomogram involving four HIPPO pathway-related genes (DLG3, TGFB3, TGFBR1, and FZD6) not merely provided a strong potential in improving prognostic stratification but exhibited essential application value for the medication decisions of gastric cancer patients, which highlighted the significance of genetics in disease prediction and drug application.

In summary, the studies included in this Research Topic have significantly enhanced the understanding of individual genetic variations, pharmacologic mechanisms, and pathogenesis underlying diseases. It is imperative for clinicians to recognize the significance of monitoring diverse drug responses, making timely dosage adjustments, and ensuring patient safety when administering medications. Although extensive research has been conducted, there’s still a pressing need for a deeper analysis of genetic susceptibility to different diseases and the impact of medication effects. Continued exploration of genetic variations, drug responses, and the interactions between diseases holds the promise of developing more precise and personalized diagnosis and treatment strategies.
